# Single-cell Raman microscopy with machine learning highlights distinct biochemical features of neutrophil extracellular traps and necrosis

**DOI:** 10.1038/s41598-023-36667-3

**Published:** 2023-06-21

**Authors:** Patrick Michael Lelliott, Alison Jane Hobro, Nicolas Pavillon, Masayuki Nishide, Yasutaka Okita, Yumiko Mizuno, Sho Obata, Shinichiro Nameki, Hanako Yoshimura, Atsushi Kumanogoh, Nicholas Isaac Smith

**Affiliations:** 1 Laboratory of Biophotonics, Immunology Frontier Research Center, Osaka University, Yamadaoka 3-1, Suita, Osaka 565-0871 Japan; 2 Department of Respiratory Medicine and Clinical Immunology, Osaka University Graduate School of Medicine, Osaka, Japan; 3 Department of Otorhinolaryngology-Head and Neck Surgery, Osaka University Graduate School of Medicine, Osaka, Japan; 4 Laboratory of Immunopathology, Immunology Frontier Research Center, Osaka University, Osaka, Japan; 5 Open and Transdisciplinary Research Institute (OTRI), Osaka University, Osaka, Japan

**Keywords:** Optical imaging, Optical techniques, Cell biology, Molecular biology, Medical research, Optics and photonics, Cell death and immune response, Imaging the immune system

## Abstract

The defining biology that distinguishes neutrophil extracellular traps (NETs) from other forms of cell death is unresolved, and techniques which unambiguously identify NETs remain elusive. Raman scattering measurement provides a holistic overview of cell molecular composition based on characteristic bond vibrations in components such as lipids and proteins. We collected Raman spectra from NETs and freeze/thaw necrotic cells using a custom built high-throughput platform which is able to rapidly measure spectra from single cells. Principal component analysis of Raman spectra from NETs clearly distinguished them from necrotic cells despite their similar morphology, demonstrating their fundamental molecular differences. In contrast, classical techniques used for NET analysis, immunofluorescence microscopy, extracellular DNA, and ELISA, could not differentiate these cells. Additionally, machine learning analysis of Raman spectra indicated subtle differences in lipopolysaccharide (LPS)-induced as opposed to phorbol myristate acetate (PMA)-induced NETs, demonstrating the molecular composition of NETs varies depending on the stimulant used. This study demonstrates the benefits of Raman microscopy in discriminating NETs from other types of cell death and by their pathway of induction.

## Introduction

Neutrophil extracellular traps (NETs) are a form of cell death characterized by breakdown of the nucleus and release of DNA in a cloud or string like structure^1,2^. Due to their delicate and varied nature, the development of specific and straightforward techniques for the characterization of NETs has been difficult^3,4^. Although a large body of research has demonstrated that NET formation is a controlled and distinct form of cell death^2,5^, significant overlap exists with other cell death pathways, and the exact definition of NETs is under debate^4^. DNA decondensation and release distinguishes NETs from processes such as apoptosis, necroptosis, and pyroptosis, all of which result in nuclear condensation^6,7^. However, later stages of many cell death pathways can result in breakdown of the nucleus and DNA release, potentially confounding endpoint analysis. For example, secondary necrosis, which occurs after apoptosis, results in DNA decondensation, cell lysis and release of extracellular contents^8–10^. Additionally, non-regulated cell death, such as necrosis caused directly by physiological damage to cells, can result in features remarkably similar to NET formation^11,12^. It is critical to distinguish these types of cell death in order to understand which pathways are being activated to cause cell death, how they can be controlled to mitigate potential pathological outcomes, and whether cell death is the result of physiological insults or stress to cells during experimental procedures or is a genuine programmed cell death pathway^6^.

NET detection and quantification usually relies on a combination of extracellular DNA measurement, cell morphology, and protein markers, the most common being myeloperoxidase (MPO), neutrophil elastase (NE), and citrullinated histones. Extracellular DNA measurement utilizes fluorescent dyes, such as PicoGreen^2^, to measure DNA released into the supernatant. Impermeable DNA dyes such as Sytox Green are also often used to stain NETs, while excluding cells with an intact membrane, allowing quantification by techniques such as flow cytometry^13–15^. While easy to implement, these techniques are limited due to their inability to distinguish NETs from other forms of cell death. Morphology analysis improves upon this, but requires imaging followed by manual counting or automated image processing^16–20^. This can be laborious and introduce biases, therefore protein markers are often introduced in an effort to improve accuracy. As well as assisting imaging techniques, protein markers are utilized in sandwich ELISAs, in which NETs are immobilized using protein antibodies, commonly anti-MPO, and detected via antibodies for DNA (or vice versa)^21–23^.

An alternative to the use of single, targeted protein markers for cell analysis is an unsupervised interrogation of total cell molecular content. This can achieve deeper levels of characterization and reveal distinguishing features not considered previously. Raman spectroscopy detects laser scattering patterns produced through molecular bond vibrations, with recent advances in label-free Raman spectral microscopy allowing the interrogation of molecular content at single-cell resolution, in live cell cultures^24,25^. The high level, complex data produced through Raman requires commensurate analysis techniques, such as dimensionality reduction through principle component analysis (PCA) or machine learning, to identify critical spectral differences between complex samples. In this way, Raman can be used to classify cell states, such as activated versus resting macrophages^24^, or cell types, such as resident versus infiltrating macrophages^26^, and cancerous versus healthy cells^27^; without requiring specific protein markers. Raman spectroscopy has seen fewer applications in studies of neutrophils, which are smaller, and can be more challenging to measure. Nevertheless, some important demonstrations of the power of Raman-based analysis have been reported: High-throughput screening of a variety of white blood cells including neutrophils^28^, with leukocyte sub-typing reporting^29^. Neutrophil activation^30,31^ and differentiation^32^ has also been tracked by Raman analysis. Aside from phenotyping and characterization of cell states, Raman spectroscopy can also shed light on more specific molecular functions in neutrophils such as lipid body associations with cellular function^33^.

In this study we used freeze/thaw necrosis, which resulted in a morphology similar to NETs, as a basis to test the ability of Raman microscopy to discriminate NETs from necrotic cells. Despite the inability of traditional techniques to distinguish these cells, we demonstrate clear spectroscopic differences in Raman signal, indicating base level molecular differences in these cell types. We use PCA to narrow down the key Raman features which separate these cell types and allow classification. We further perform analysis utilizing logistic regression-based machine learning to show that NETs produced by different stimuli can be distinguished by their Raman spectroscopic fingerprint. Overall, we provide a deeper understanding of the differences between necrosis and different types of NET formation, highlighting molecular differences which can be clearly identified through Raman microscopy, thereby providing an important approach for NET analysis.

## Results

### Limitations of DNA morphology and single protein marker approaches in distinguishing necrosis from NET formation

We first assessed the ability of conventional techniques to distinguish NETs from necrotic neutrophils. We induced NETs using two model stimuli, phorbol myristate acetate (PMA) and lipopolysaccharide (LPS), while to induce necrosis we used one round of freeze/thaw at − 80 °C, which we found produced necrotic cells similar in appearance to NETs. We analyzed cells using immunofluorescence microscopy, PicoGreen DNA quantification, and DNA/MPO complex ELISA, according to widely adopted protocols.

**Figure 1:**
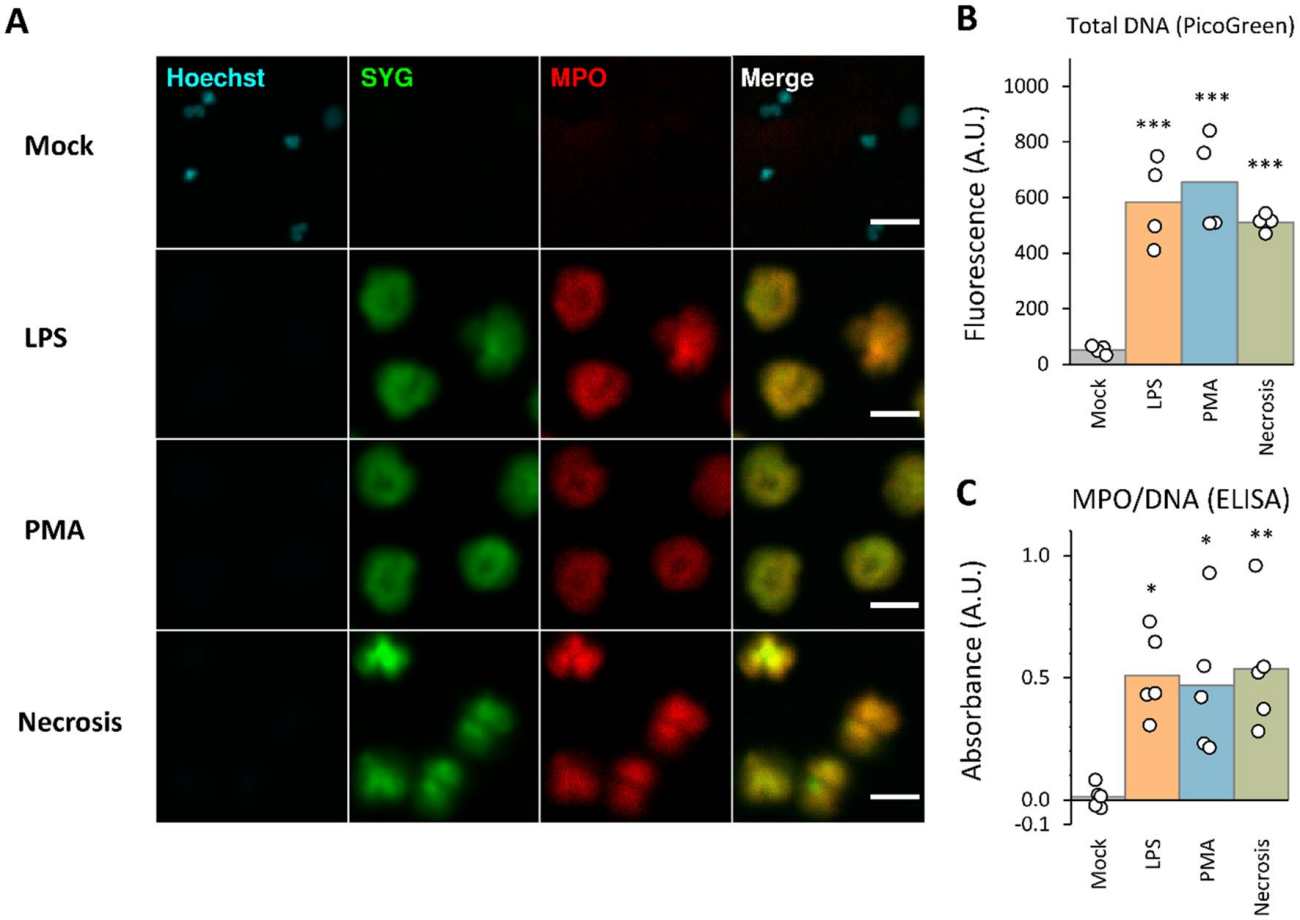
Limitations of DNA morphology and single protein marker approaches in distinguishing necrosis from NET formation. Representative images of mock treated neutrophils, LPS-induced NETs (10 μg/ml), PMA-induced NETs (100 nM), and necrotic cells (freeze/thaw) stained with Hoechst (cyan), Sytox Green (SYG, green), and PE conjugated anti-myeloperoxidase antibody (MPO, red) after 4 h incubation at 37 °C, imaged directly in plate wells without washing (**A**). Note that the Hoechst intensity is optimized for visualization of the mock sample, making its signal indiscernible for NETs and necrotic cells which have a much lower DNA density. Scale bar is 25 μm. DNA collected by initial washing, and then lifting from the surface through partial nuclease digestion, was quantified by PicoGreen fluorescence for total DNA (**B**), and by anti-MPO capture, anti-DNA detection ELISA for MPO/DNA complexes (**C**). Data points represent independent experiments on different days with different donors. ***p < 0.001, **p < 0.01, *p < 0.05, as determined by ANOVA with Tukey post hoc test.

For immunofluorescence microscopy, we used permeable and impermeable DNA dyes Hoechst and Sytox Green, combined with a fluorescent anti-MPO antibody (Fig. 1A). NETs presented as large diffuse cloud like structures of DNA, which co-localized with MPO staining, with no immediately evident differences between PMA and LPS-induced NETs. Necrotic cells displayed a similar cloud like DNA morphology, and critically, necrotic cells also stained robustly with MPO co-localized with DNA. An isotype control for the MPO antibody used gave little signal, indicating non-specific adsorption of antibody is unlikely (Supp Fig. 1A). Automated masking and quantification of cell area, and Sytox Green and MPO staining intensity (cell features commonly used for NET analysis), showed that although there were significant differences between groups, these features largely overlapped between NETs and necrotic cells (Supp Fig. 2).

Next, we used PicoGreen fluorescence, an indicator of NET formation based on extracellular DNA release. This assay can be performed on DNA released directly into the supernatant, or DNA released from the surface after agitation and/or partial nuclease digestion. Preliminary experiments indicated partial digestion using micrococcal nuclease gave the most consistent results and therefore we adopted this approach. As indicated by PicoGreen fluorescence intensity, necrosis and both types of NET induction resulted in a robust release of DNA compared to mock, but there were no significant differences across the treated groups (Fig. 1B). This indicates DNA from freeze/thaw necrosis remains attached to the surface during washing and is released by nuclease digestion in a similar way to NETs.

Finally, we analyzed samples using DNA/MPO complex ELISA. This approach is similar to the PicoGreen approach, however, is generally considered to be a more specific marker of NET formation given that only DNA/MPO complexes are measured, rather than total DNA. Despite this, necrotic cells and NETs produced equivalent levels of DNA/MPO complexes (Fig. 1C). To address any potential non-specific adsorption an isotype control was used in place of the anti-MPO capture antibody, however, no appreciable signal was detected (Supp Fig. 1B). Overall, we demonstrate that distinguishing NETs from freeze/thaw necrotic cells is problematic using conventional techniques.

### Single-cell Raman measurements of NETs and freeze/thaw necrotic cells using a multimodal optical platform provide a molecular overview of cellular content

**Figure 2:**
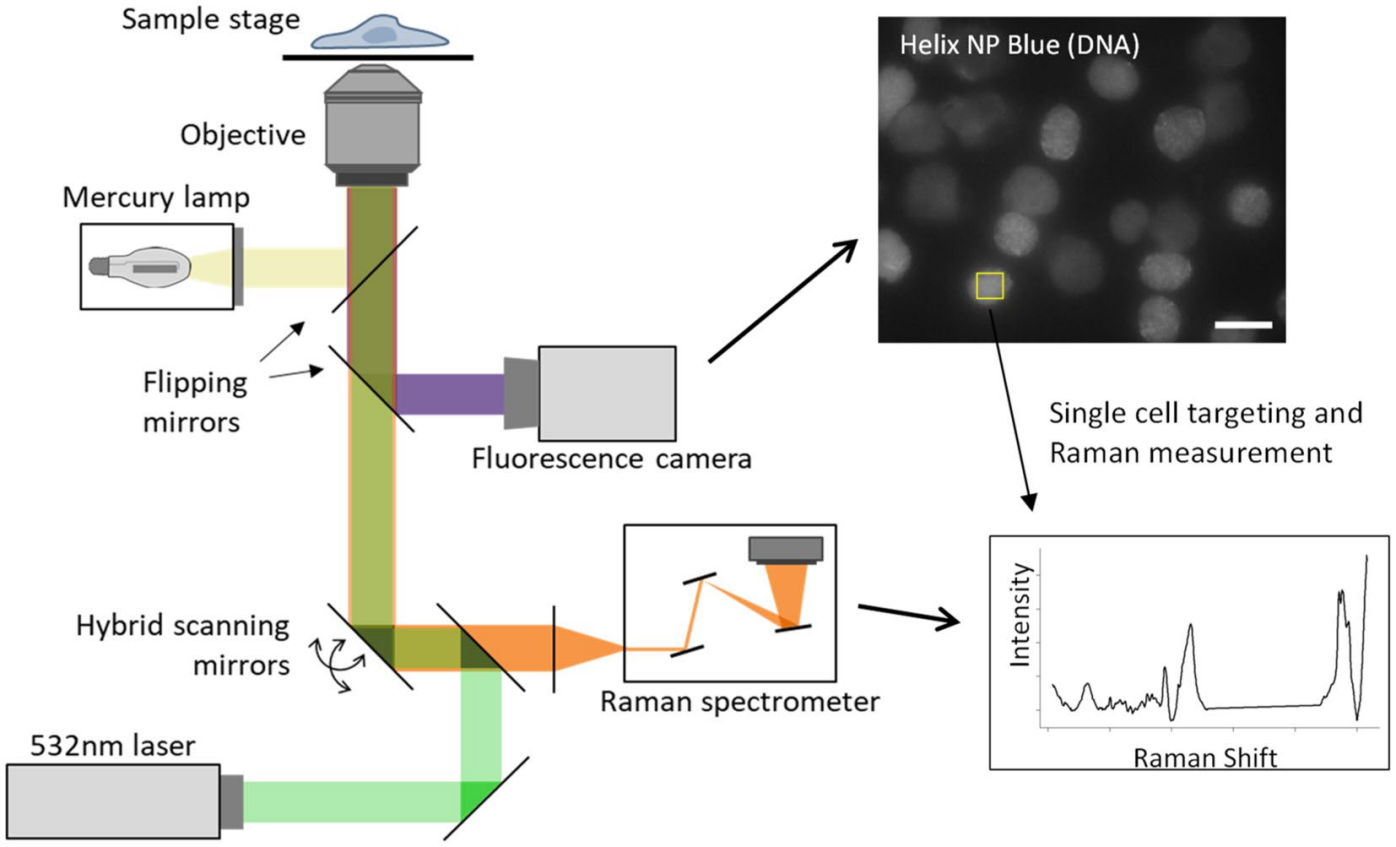
Single-cell Raman measurements of NETs and freeze/thaw necrotic cells using a multimodal optical platform. Cells are spatially located using the fluorescent marker Helix NP Blue. Scale bar is 25 μm. Flipping mirrors are then used and a laser is rapidly moved across the central area of a single cell using hybrid scanning mirrors, from which the Raman scattering spectrum is collected.

As shown above, we demonstrated that identification of NETs using DNA morphology or a single protein marker can be challenging. An alternative approach is to perform an overall study of molecular cell features, which we achieved using Raman microscopy. NETs and necrotic cells were induced in the same way as above. Cells were interrogated used a custom-built optical platform combining fluorescence and Raman spectroscopy that we have previously described^24^ (Fig. 2). Due to their limited brightfield contrast it was not possible to identify the location of unlabeled NETs, and it was therefore necessary to include a DNA fluorescence marker, Helix NP Blue, which we confirmed did not interfere with Raman signal (Supp Fig. 3). NETs and necrotic cells were identified based on a large area and low intensity Helix NP Blue signal, indicating a diffuse cloud of DNA typical of NETs.

**Figure 3:**
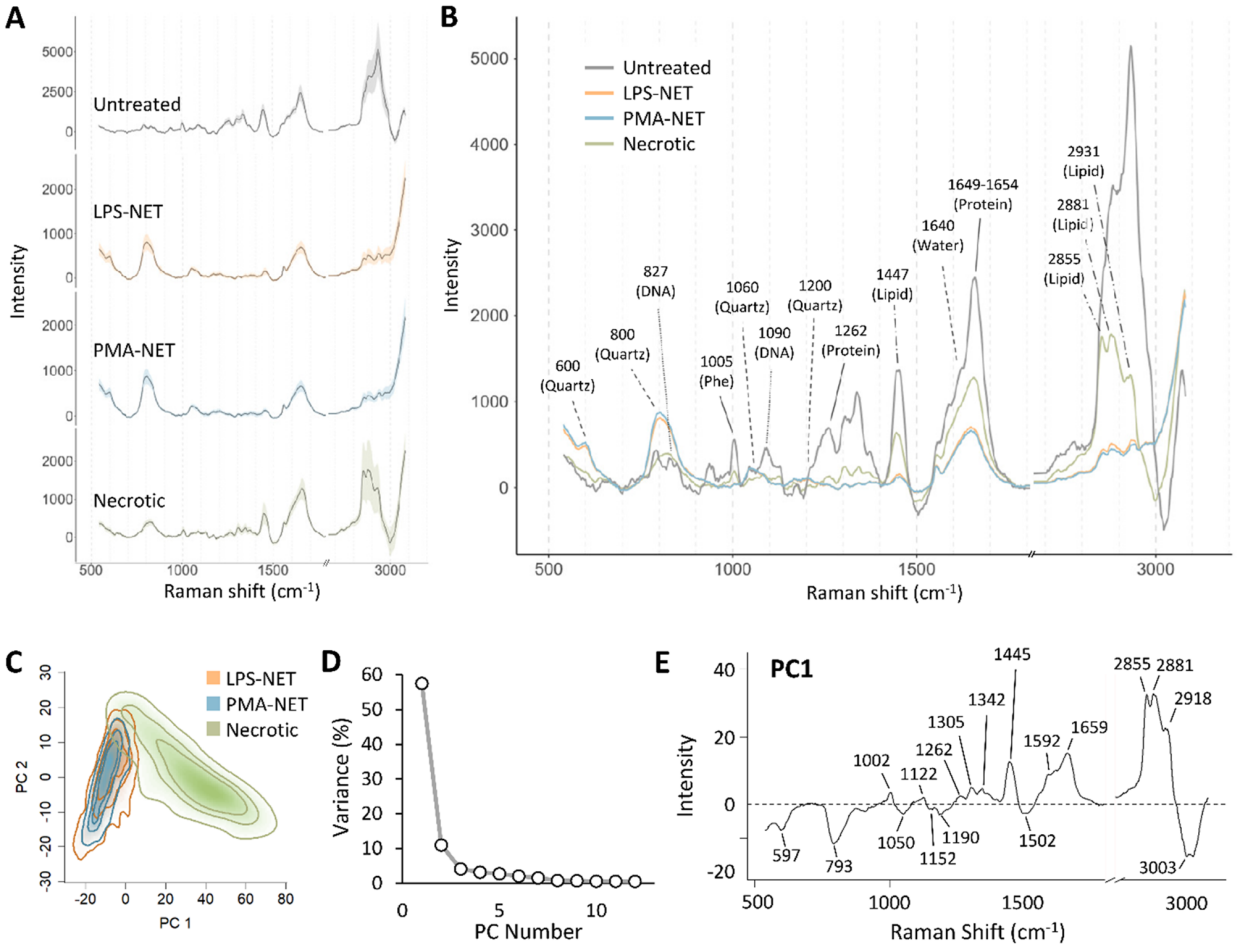
Principal component analysis of Raman spectrographic features highlights the major molecular differences between necrotic cells and NETs. Average, baseline-corrected single-cell Raman spectra for each treatment group, plotted separately with standard deviation represented by the shaded region (**A**), or overlaid on one plot (**B**). Scores plot (**C**), variance explained by each component (**D**), and loading vector for PC1 (**E**) for principal component analysis (PCA) of LPS, PMA, and necrotic cell Raman spectra.

The average Raman spectra, baseline-corrected by cubic spline, are shown from each treatment group (Fig. 3A,B). Due to baseline correction, some portions of the spectra appear negative, however peak positions and strengths are comparable across the datasets. The resulting spectra are typical of live immune cells, with the spectra primarily consisting of protein and lipid related bands, with some smaller contributions from other cellular molecules such as DNA and RNA. More detail on the origins of these bands are given in Table 1. The average Raman spectra of the LPS and PMA-induced NETs are almost identical to each other, showing a marked loss of biological content compared to untreated cells, while the average Raman spectrum of the necrotic neutrophils also shows a notable loss of biological content albeit not as severe (Fig. 3A,B). Major differences between necrotic cells and NETs include a reduction in the contribution from the quartz substrate, and increased protein and lipid contributions. Overall, results suggest that when forming NETs, neutrophils undergo a dramatic loss of cellular content, with the remaining material contributing to the Raman spectra predominantly protein-based. Necrotic cells likewise lose a significant amount of the material contributing to their Raman spectra, but appear to retain more of their lipid content.

**Table 1:** Raman band assignments.

Un-treated	LPS	PMA	Necrotic	3 way PCA PC1	2 way PCA PC1	2 way PCA PC2	2 way PCA PC5	Assignment
	600	600		597 (−)	600 (−)	600 (−)		Fused quartz^57^ (sample carrier)
666								
728								
751								
790	802	799		793 (−)	799 (−)	800 (−)		Fused quartz^57^ (sample carrier)
827			821					Nucleic acid backbone^58,59^
							877 (−)	H-bonding indole ring tryptophan^35^
935								Protein backbone^34^
1002			1005	1002 (+)				Phenylalanine^34,35,60^, proteins^61^,
1046	1049	1052		1049 (−)				Protein backbone stretch^34^
					1065 (−)	1064 (−)		C–C and C–N stretching^62,63^, saturated lipids^63^, possible contributions from fused quartz^57^ (sample carrier)
			1084				1087 (−)	Protein backbone stretch^34^, PO_2_ symmetric stretch phospholipids^35^, (possible additional contributions from C–C and C–N stretching saturated lipids^62,63^)
1092								Nucleic acid backbone^58,64^
1122			1127	1122 (+)	1122 (+)			Lipids^61^, C–C and C–N stretching^62,63^, saturated lipids^63^
				1152 (−)				
	1165	1165	1165					C–C and C–N stretching^62^
1173								Tyrosine and phenylalanine^60^
				1190 (−)				
	1203	1203	1206					Fused quartz^57^ (sample carrier)
	1254	1254						Amide III random^60^, Amide III polyproline II helix^34^, protein^61^, Amide III^62^, Amide III β-sheet^35^, PO_2_ asymmetric stretch phospholipids^35^
1259			1262	1262 (+)				Amide III α-helix/β-turns^34^, Amide III C–H bending and C–N stretching^65^, Amide III disordered^35^, C=CH_2_ bending in lipids^61^, H–C= in plane deformations lipids^33^
1307	1304	1307	1305	1305 (+)				Lipids^61^, CH_2_ twisting of (saturated) hydrocarbon chains^63^, CH_2_ wagging^62^, Amide III C–H bending and C–N stretching^35,65^
1336								CH_2_ deformation^60^, Amide III α-helix^34,35^, protein^61^, C–H and tryptophan^62^
	1345	1342	1342	1342 (+)				CH_2_ deformation^60^, Amide III α-helix^34,35^, protein^61^, C–H and tryptophan^62^
1373	1373	1373						CH_3_ stretching^62^
	1389	1389						
1447	1452	1452	1447	1445 (+)		1447 (−)		CH_2_ and CH_3_ deformation^33,60,62,63^, lipid and protein contributions^61^
							1458 (−)	N–H bending, C–N stretching^34^, CH_3_ H–C–H deformation^66^
				1502 (−)				Amide III^62^
1556	1559	1559	1559					Indole ring breathing^34^, Tryptophan, C-H bending and C–N stretching^65^
1587				1592 (+)				
1620								Tyrosine^34,60^
	1649	1649			1646 (+)			Α-helix, disordered structure^67^, some contributions from water
1656			1654	1659 (+)				Amide I helix^60^, α-helix^34,67^, disordered^67^, Amide I^62^, H–C=O stretch Amide I α-helix^35^, C=C vibrations unsaturated lipids^33,66^
	2859	2859	2855	2855 (+)		2855 (−)	2855 (+)	CH_2_ symmetric stretching^62,66,68^
2883	2881	2878	2881	2881 (+)		2881 (−)	2883 (−)	CH_3_ symmetric stretching^68^, Saturated lipid bonds^35^
				2918 (+)				CH_2_ stretching (close to CH_3_ or carboxyl residue)^66^
2931			2931			2929 (−)	2929 (−)	CH_2_ asymmetric stretching^68^
	2938	2938						CH_2_ asymmetric stretching^68^, Saturated lipid bonds^35^
				3003 (−)				H–C= stretch in lipids^33^, H–C=C stretch unsaturated lipids^35^
						3029 (+)		CH3 asymmetric stretching y^68^

(+) and (−) represent the direction of the band in the PCA loading vector.

### Principal component analysis of Raman spectrographic features highlights the major molecular differences between necrotic cells and NETs

To explore the major contributions to variance in the dataset we preformed PCA of Raman spectra obtained from necrotic cells and NETs. The largest amount of variance in the dataset (PC1, 57%) divides the data into two clearly separated populations consisting of necrotic cells, with largely positive PC1 values, and NETs, giving negative values (Fig. 3C,D). The loading vector for PC1 is shown (Fig. 3E) with candidate spectral contributions summarized in Table 1. Overall, compared to necrotic cells, LPS and PMA-induced NETs have greater contributions from quartz as well as from the protein backbone (band at ~ 1049 cm^−1^)^34^ and H–C=C vibrations (band at ~ 3003 cm^−1^)^35^ suggesting the presence of unsaturated fatty acid chains are more strongly associated with NETs.

**Figure 4:**
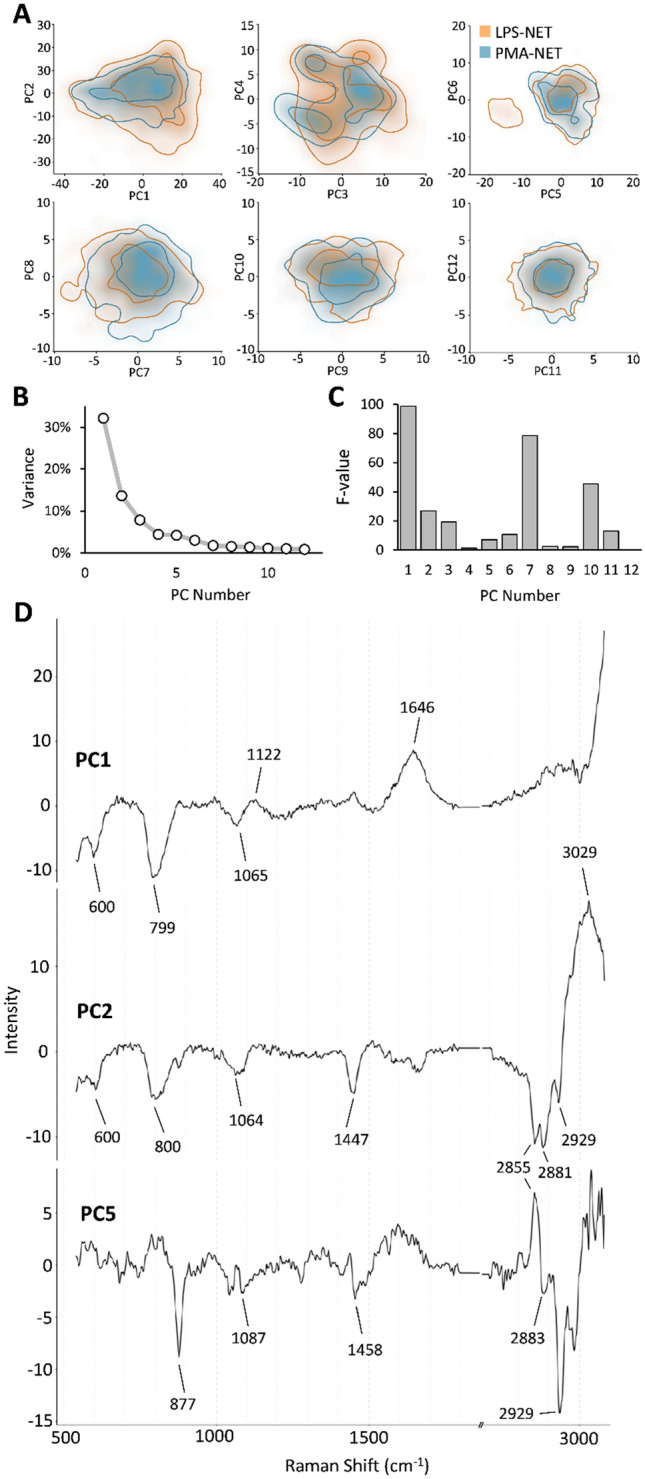
Principle component analysis was unable to distinguish PMA and LPS-induced NETs. Scores plot (**A**), and variance explained by each component (**B**) for principal component analysis (PCA) of LPS and PMA-induced NETs Raman spectra. Contour lines represent the 50th and 90th percentiles of the dataset, with N = 1332 NETs from LPS and N = 1458 NETs from PMA treated cells. F-test values per treatment group indicating separating power for each PC (**C**). Loading vectors for PC1, PC2, PC5, with other PCs shown in Supplementary Fig. 4.

To specifically explore differences between LPS and PMA induced NETs, a second PCA was performed with the necrotic data excluded. While the PC scores plots show it is difficult to separate the two treatment types, some small differences exist in PC1, PC2, and PC5 (Fig. 4A,B) To calculate the separating power between treatment groups for each PC, we calculated F-values using ANOVA as previously described^26^ (Fig. 4C). PC1 was highest, indicating that this PC provides the best overall separation of the two treatment groups. PC7 and PC10 displayed the next highest F-values, and we plotted these against PC1 (Supp Fig. 4), however these PCs were very similar between the two groups and their loading vectors were not biologically informative (Supp Fig. 5). Loading vectors for the PCs explaining most of the variance and population differences, PC1, PC2, PC5, are shown (Fig. 4D), with band contributions summarized in Table 1. Overall, due to the spectral similarity between LPS and PMA NETs, PCA was not able to distinguish between the two populations.

### Machine learning reveals molecular differences which discriminate LPS and PMA-induced NETs

To determine if there are fine molecular differences between LPS and PMA-induced NETs detected in the Raman spectra not picked up in the PCA analysis, we used supervised machine learning based on a penalized logistic regression model^24,26^. This method uses the baseline-corrected spectra, but not PCA-processed spectra. It attempts to identify only the most useful spectral parameters based on the accuracy of classification using a labelled training dataset. Parameters which do not improve classification accuracy significantly are removed using the lasso approach, which reduces these parameters to zero by introducing a penalty term into the algorithm.

**Figure 5:**
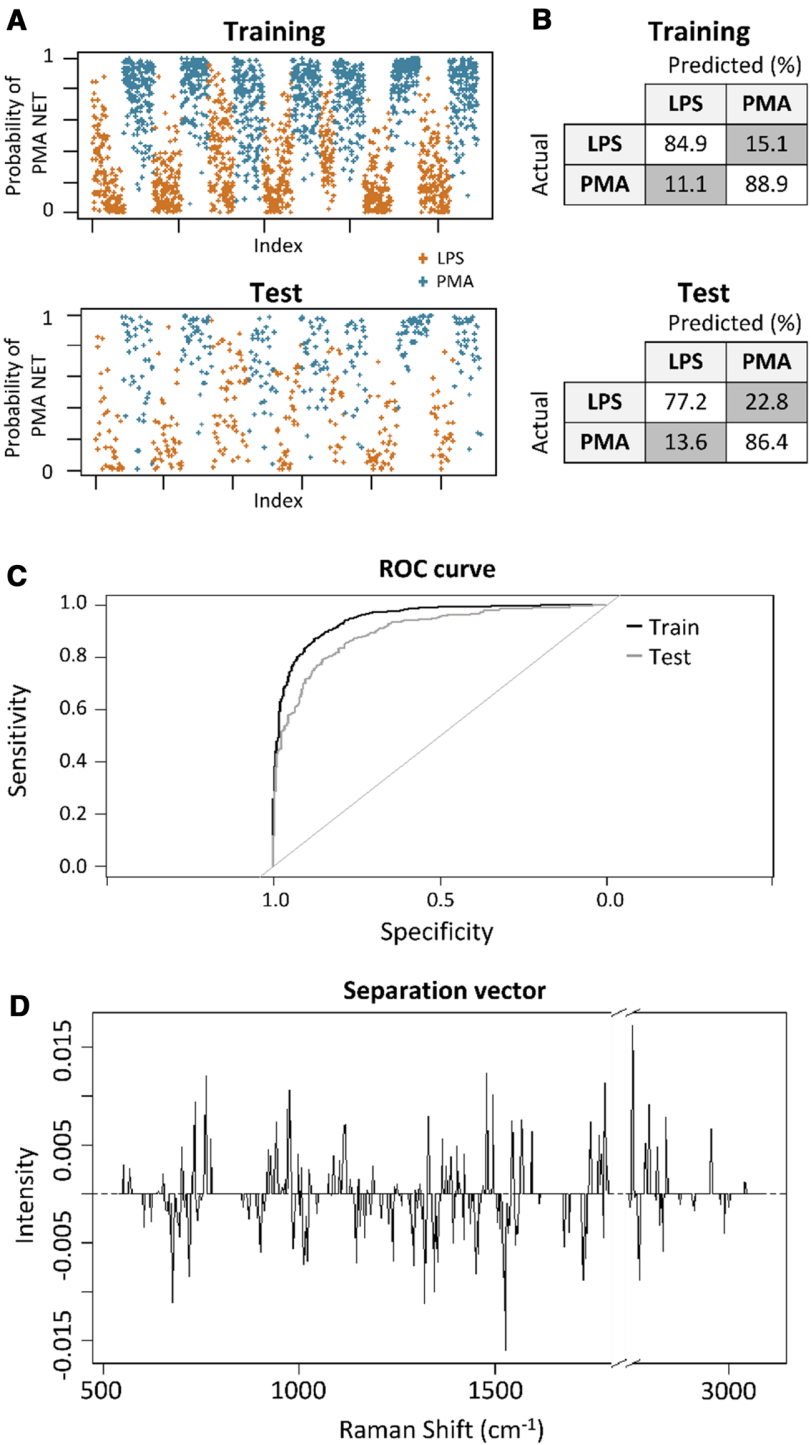
Machine learning reveals molecular differences which discriminate PMA and LPS-induced NETs. Probability scores for each cell across the training and test dataset (**A**). Confusion matrices showing prediction accuracy (**B**), and ROC curve showing specificity and sensitivity of the model (**C**). Separation vector used for classification (**D**). For the training dataset, N = 1060 for LPS treated and N = 1172 for PMA treated. For the test dataset, N = 272 for LPS treated and N = 286 for PMA treated.

Data was randomly split into a training set comprising of 80% of samples in which treatment group information is provided to the model, and a test set containing the remaining 20% from which the model attempts to classify samples without a priori knowledge of their treatment. The model accurately identified 77% and 87% of LPS and PMA-induced NETs respectively in the test dataset (Fig. 5A–C), which indicates that subtle but consistent molecular differences exist between NETs, and that these differences can be reliably detected in our Raman measurements. The spectral features used in the model are depicted by the separation vector (Fig. 5D). Despite attempts by the model to perform parameter reduction, the separation vector remains highly complex and difficult to interpret. This indicates that classification requires a large set of molecular information to tease out the consistent differences in the two treatment groups. Overall, we demonstrate that despite very similar spectra in LPS and PMA-induced NETs, there are nevertheless complex, small-scale differences between these treatment groups which can be picked up by fine tuning classification through supervised machine learning.

## Discussion

The need for techniques which unambiguously distinguish NETs from other forms of cell death was listed as one of four key issues in NET research in a recent panel review^4^. This study highlights the utility of Raman microscopy in discriminating molecular differences between NETs and necrotic cells, as well as between NETs produced by different stimuli. We demonstrate that these subtle differences can be difficult to detect using conventional methods, such as immunofluorescence microscopy or ELISA, emphasizing the limitations of using select biomarkers for NET quantification, and the benefits of a holistic approach such as Raman microscopy.

Before applying a Raman approach, we tested the ability of three common techniques to distinguish NETs from necrotic cells: DNA quantification with PicoGreen, MPO/DNA ELISA, and immunofluorescence microscopy. Standard protocols for PicoGreen and MPO ELISA assays include an initial washing step, to remove degraded and necrotic DNA, followed by partial nuclease digestion to release the NET DNA remaining attached to the substrate^22^. We followed this protocol, however compared to NET formation basic freeze/thaw-induced necrosis resulted in similar levels of DNA retained on the substrate and released during nuclease digestion. Importantly, this held true for DNA detected directly through PicoGreen fluorescence, or in complex with MPO and detected through ELISA. Interestingly, The DNA binding properties of MPO have been described^36^, and it seems plausible that MPO would naturally form a complex with DNA when these molecules come into contact. In support of this, direct disruption of neutrophil cell membranes through electropermeabilization results in the release of MPO in a complex with DNA resembling NETs^12^. In contrast, other studies indicate that apoptotic neutrophils induced by TNF-α^21,23^, and necrotic neutrophils induced by sonication^21^, do not result in measurable MPO/DNA complex formation. One reason for this could be that these forms of cell death do not release significant amounts of DNA outside the cell, or in the case of sonication, perhaps DNA/MPO complexes are disrupted. Another plausible explanation is that in our protocol, necrotic cells were incubated at 4 h at 37 °C (the same amount of time as NET induction), and perhaps this time is required for MPO to form a complex with DNA.

We also examined cells by immunofluorescence microscopy. While the morphology and staining intensity of necrotic cells and NETs was quite similar, including staining with fluorescent MPO antibody, there were subtle differences which could be picked up on when comparing them side by side. While these could feasibly be used to discriminate NETs accurately, they would be subject to bias and variations in sample preparation^4,37^, and current automated techniques based largely on size and extent of Sytox green staining would not likely be sophisticated enough to classify cells based on these differences^17,18,38–40^. Deep learning algorithms are able to hone in on subtle image differences to classify cells, and have been successful in distinguishing freeze/thaw necrotic cells, similar to those presented here, from NETs^11^. These models are still in the early stage of development, and rely on large, consistent datasets. Given the aforementioned variability in NET morphology, it remains to be seen if these will be feasible as a general technique to quantify NETs.

In contrast to conventional techniques, Raman microscopy could clearly detect differences between NETs and necrotic cells. Simple PCA analysis, which relies only on intrinsic data variance in an unsupervised way, was able to separate these cell types into two distinct populations based on Raman signal. This allows the discrimination of NETs from necrosis which is unbiased and highly specific, without employing complex algorithms based on deep learning, and without relying on staining of potentially non-specific protein markers. It is not possible to extract the exact molecular differences from the complex, intermingled Raman spectrum of each cell type, however, some overarching differences can be examined. Necrotic cells seem to contain relatively higher amounts of cellular material compared to NETs, in particular, they contain higher levels of lipids. This is an indication that NET formation results in a more complete disassembly of the cell membranes, which is in keeping with the biological mechanisms known to be required for NET formation such as the activity of Gasdermin D^41^. Notably, although DNA is generally considered as the main component of NETs, the actual DNA density is far less than that contained within a cell nucleus, and ultimately this lack of density results in a Raman signal below the limit of detection in our system. Overall, owing to their diffuse nature, the Raman signal produced by NETs is relatively low. Despite this our system is able to detect clear differences between NETs and freeze/thaw necrotic cells, which are difficult to distinguish using standard imaging and biochemical methods. Taking this one step further, our system was also able to distinguish NETs based on their pathway of induction.

NETs induced by PMA and LPS produced markedly similar Raman spectral signatures. The differences between these were too small to be picked up by PCA. Supervised learning approaches, in which algorithms are directed to actively differentiate two classes, offer a much more powerful approach to classify cell types. Our logistic regression model was able to achieve a high level of accuracy on a test dataset, correctly categorizing cells approximately 80% of the time and indicating that there are subtle but consistent biological differences between PMA and LPS-induced NETs. Consistent with this, proteomics studies of NETs have shown differences between PMA and LPS-induced NETs^42^. PMA has been criticized for a lack of biological relevance, and our data supports the idea that PMA NETs are measurably different to those induced by LPS. It is possible these differences would affect the activity of NETs on other cells and tissues.

A limitation of this study is that we only collected data for necrosis on non-programmed cell death (through freeze/thawing cells), rather than programmed cell death, such as apoptosis. There were two reasons for this. Firstly, we wished to obtain a population of cells which displayed similar properties and appearance to NETs, but were clearly not a type of NET formation. Most types of programmed cell death involve condensation of DNA and morphology which can be easily distinguished from NETs, although this is less the case in secondary necrosis, whereby dead cells can start to break down and resemble NETs more closely^8–10^. Secondly, the exact pathways and definition of NETs are still debated^4,43^, and neutrophil responses to stimuli can be heterogenous. Many types of stimulants reported to induce a particular type of cell death have also been reported to induce NETs. For example, TNF-α induces neutrophil apoptosis^44^, but has also been reported to induce NETs^45^. It is not clear if the NETs reported from TNF-α stimulation are apoptotic cells that have undergone secondary necrosis, or if secondary necrosis should be considered a type of NET formation under current definitions^6^.

Overall, we demonstrate that with a sufficiently sensitive system, it is possible to collect single cell Raman spectra from NETs which can act as a molecular fingerprint for each cell. This collection is minimally invasive, requires little sample preparation, and is relatively high throughput and low cost. Unlike classical techniques used for NET quantification this technique easily discriminates necrotic cells from NETs, highlighting the clear molecular differences resulting from the distinct process of NET formation as opposed to unregulated cell death. Deep exploration of the complex Raman information collected from cells using machine learning can reveal subtle molecular differences in NETs determined by their route of induction. These molecular signature differences point to a fundamental difference between these NETs which could bear significance in future studies related to downstream consequences of NET formation and other areas.

## Materials and methods

### Blood collection and neutrophil isolation

Blood samples were collected from three healthy volunteers across seven experiments (two times from donor A, four times from donor B, and one time from Donor C) after obtaining informed consent in accordance with the Declaration of Helsinki with approval from the ethical review board of the Graduate School of Medicine, Osaka University, Japan (no. T19204 and no. 11122-5). Neutrophils were isolated using EasySep Direct Human Neutrophil Isolation Kit (Stemcell Technologies, Vancouver, Canada).

### NET induction

Experiments were performed in Dulbecco’s Modified Eagle Medium: Nutrient Mixture F-12 (DMEM/F12) with 15 mM HEPES, without phenol red (Thermo Fisher Scientific, Waltham, MA) supplemented with 0.005% human serum albumin (HSA, Sigma-Aldrich, St. Louis, MO). To induce NETs, neutrophils were incubated with 100 nM PMA or 10 μg/ml LPS from Escherichia coli O128:B12 (Sigma-Aldrich) at 37 °C for 4 h with 5% CO_2_. Note that LPS-induced NET induction is highly sensitive to HSA concentration and needs to be optimised for LPS strain and incubation buffer^46,47^. For necrosis, suspended neutrophils were frozen at − 80 °C, then defrosted at room temperature, before aliquoting to plates or dishes and incubation at 37 °C for 4 h with 5% CO_2_. The percentage of NET formation varies from donor to donor and between treatments. For PMA the NET percentage is 90–100%, while for LPS it is 30–90%. Spectra were only collected from NETs.

### Immunofluorescence microscopy

Immunofluorescence Microscopy was performed as previously described^48^. Cells were seeded in flat-bottom 96-well tissue culture-treated plates (Thermo Fisher Scientific) at 6 × 10^4^ cells/cm^2^. Cells were blocked with 0.5% HSA before staining with 4 µM Hoechst 33342 (Sigma-Aldrich), 500 nM Sytox Green (Thermo Fisher Scientific), and 20 μg/ml PE conjugated mouse IgG1 anti-human MPO (Clone: MPO-7, Agilent Technologies, Santa Clara, CA) or 20 μg/ml PE conjugated mouse IgG1 isotype control (Clone: MOPC-21, Biolegend, San Diego, CA) for 30 min at room temperature. Imaging was performed within wells using a CQ1 fluorescence microscope with 10× objective (Yokogawa, Tokyo, Japan).

### NET and necrotic DNA isolation

Cells were seeded in flat-bottom 96-well tissue culture-treated plates at 3 × 10^5^ cells/cm^2^. After treatment, supernatant was removed and discarded from wells, and cells were incubated with 0.5 U/ml micrococcal nuclease (New England Biolabs, Ipswich, MA) for 10 min at 37 °C. DNA digestion was stopped by the addition 5 mM EDTA with a final cell concentration of 1.4 × 10^6^ cells/ml. Cells were vigorously pipetted, then centrifuged for 5 min at 500×*g*. Supernatant was collected and frozen at − 80 °C.

### Total DNA assay and MPO/DNA ELISA

For the total DNA assay, NET/DNA samples were stained with PicoGreen and fluorescence read on GloMax microplate reader (Promega, Madison, WI). The MPO/DNA ELISA was performed as previously described^49^. Briefly, 96-well flat bottom Nunc MaxiSorp plates (Thermo Fisher Scientific) were coated with 5 μg/ml mouse IgG2b anti-human MPO (Clone: 4A4, Bio-Rad, Hercules, CA) or 5 μg/ml mouse IgG2b isotype control (Clone: 27–35, Biolegend) for 1 h at room temperature. Remaining steps were performed according to the Roche Cell Death Detection ELISA kit (Cat. No. 11544675001, Sigma-Aldrich).

### Raman microscopy

Cells were seeded in 3.5 cm quartz bottom dishes (Matsunami Glass, Osaka, Japan) at 6 × 10^4^ cells/cm^2^. After treatment, cells were stained with 1 μM Helix NP™ Blue for 30 min and analysed using a 60 × (NA 1.27) objective on a custom optical platform we have previously described in detail^50,51^, depicted in Fig. 2. Fluorescence images acquired using mercury lamp excitation and FITC channel filtered emission were used to locate NETs and necrotic cells. Raman excitation was performed with a continuous-wave 532 nm laser (1136 mW/μm^2^), with back-scattered light directed to a spectrometer using a dichroic mirror, and the vibrational spectrum (535–3075 cm^−1^) measured with a cooled camera (Orca 4.0, Hamamatsu, Shizuoka, Japan). The focus was set according to the visibility of the cell features, at roughly 2 μm above the substrate. Cells were interrogated using hybrid scanning, in which the laser excitation point is rapidly moved on the vertical and horizontal axes illuminating a defined square central region of each cell, with the average signal collected. Data were collected for 700–1100 cells per treatment group, pooled from seven independent experiments and three different donors.

### Data analysis and statistics

Raman data was corrected for day-to-day instrument spectral shifts using a standard ethanol spectra, baseline corrected using a cubic spline, and the silent region (1800–2700 cm^−1^) removed as previously described^26^. Briefly, PCA, machine learning analysis, plotting, and statistics calculations were performed using R v3.6.1^52^. Data were standardized and PCA performed using the *prcomp* function, and F-values calculated by ANOVA using the *aov* package. Machine learning was performed using a penalized logistic regression model with lasso based parameter reduction implemented with *glmnet*^53^, with the penalization term optimized using tenfold cross validation. Spectra and PC plots were created using *ggplot2*^54^, *ggbreak*^55^, and *pROC*^56^ packages. Total DNA assay and MPO/DNA complex ELISA data were analysed by ANOVA with post hoc Tukey’s tests using the *aov* package. A p-value < 0.05 was considered significant.

## Supplementary Information

Supplementary Figures.

## Data Availability

The datasets used in the current study are available from the corresponding author on reasonable request.
