# Supplementary material for: Single-cell Raman microscopy with machine learning highlights distinct biochemical features of neutrophil extracellular traps and necrosis
Source: Sci Rep. 2023 Jun 21;13:10093. doi: 10.1038/s41598-023-36667-3 (PMC10284916; doi:10.1038/s41598-023-36667-3)
Supplement: Supplementary file 1 — Supplementary Figures. [file 41598_2023_36667_MOESM1_ESM.pdf]

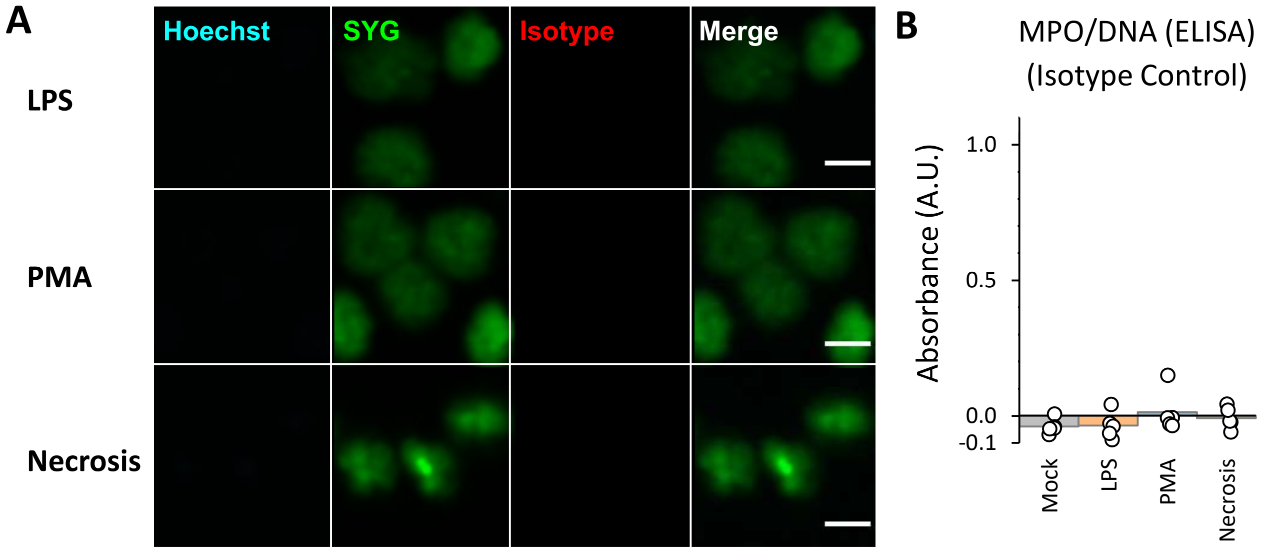

Supp Figure 1. Isotype control for anti-myeloperoxidase staining and ELISA.

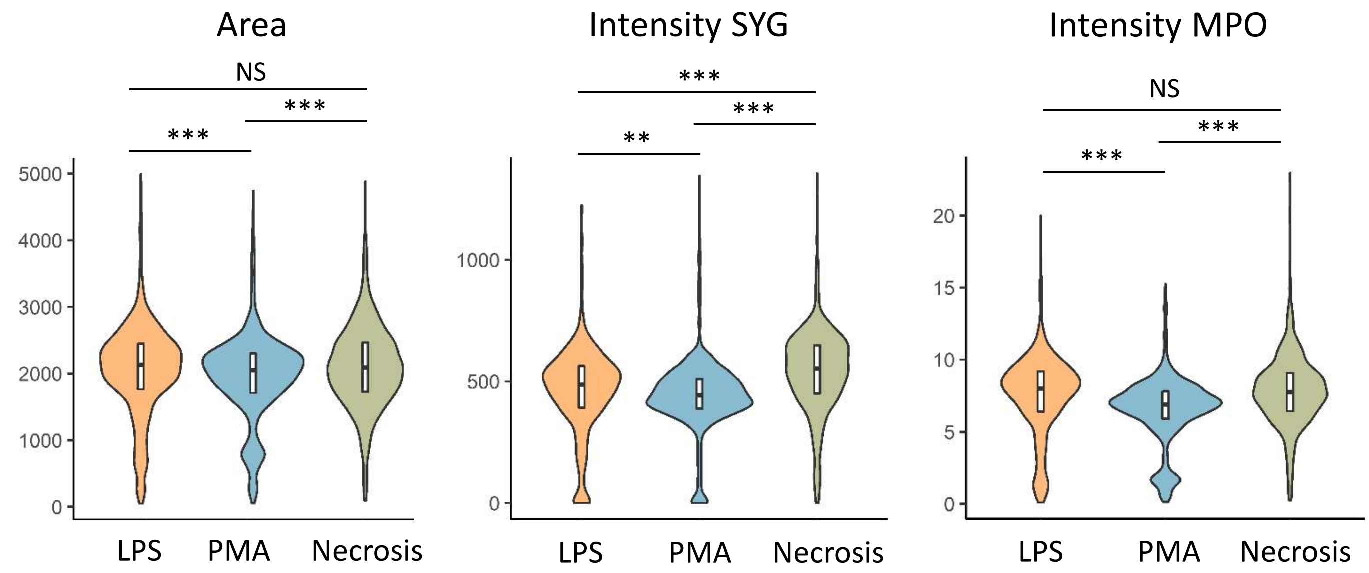

Supp Figure 2. Automated masking and feature measurement of microscopy images using a CellProfiler pipeline. LPS (N = 1423), PMA (N = 1286), Necrosis (N = 1529), *** $p < 0.001$, ** $p < 0.01$, * $p < 0.05$, as determined by ANOVA with Tukey post hoc test.

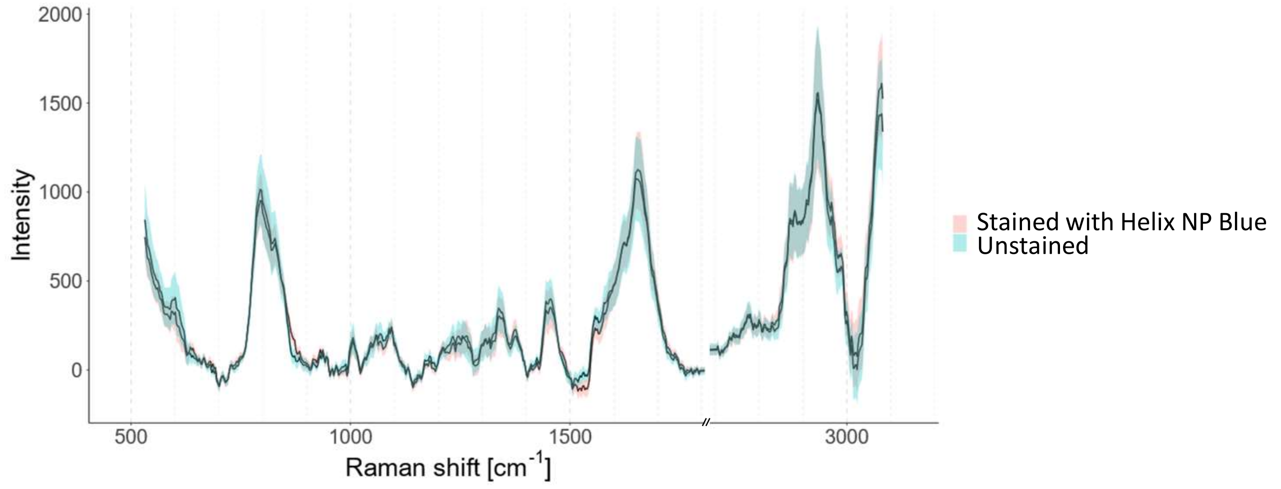

Supp Figure 3. Average Raman spectra of fixed and permeabilized neutrophils left unstained or stained with Helix NP Blue, with standard deviation represented by the shaded region.

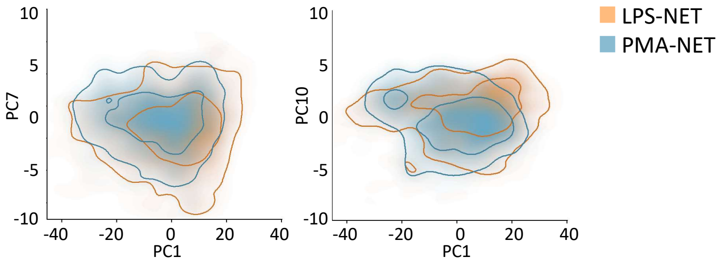

Supp Figure 4. Principle component analysis scores plot for PC1, PC7, and PC10.

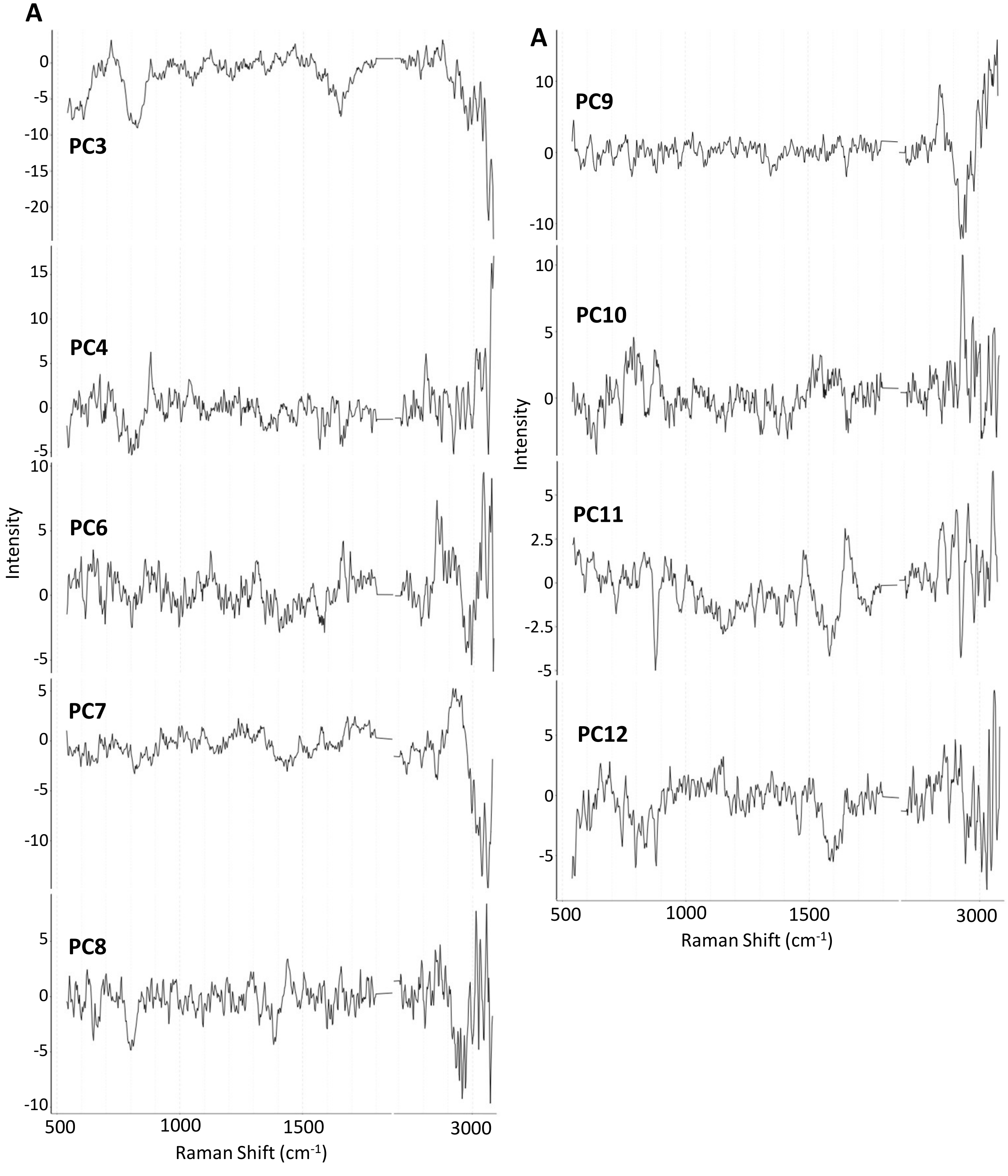

Supp Figure 5. PC loading vectors from LPS and PMA-induced NETs PCA of Raman spectra.
